# Medical Items

**Published:** 1908-11

**Authors:** 


### MEDICAL ITEMS

The fall season brings cool weather and raw winds. This condition checks elimination through the skin. More work is thrown upon the kidneys. It is not always that they are equal to the extra task imposed. Imperfect elimination is the result. The autotoxic state which soon develops is expressed in either so-called gouty bronchitis, with or without asthma, gouty eczema, recurrent tonsillitis, or rheumatism. To establish adequate elimination is to remove the cause and thus effect a rational cure. The ideal eliminant in such cases is Alkalithia, made by the Keasbey & Mattison Co., Ambler, Pa.

---

We wish to acknowledge the receipt of the handsome booklet of Howell Park Sanatarium. Three years ago, Dr. J. Cheston King established this institution for the treatment of nervous and mild emntal diseases, and by his untiring energy and close application to his special line of work, he has enlisted the interest of the medical profession and the patronage of his Sanitarium is not confined to the South alone..

### THE LOCAL TREATMENT OF CATARRHAL CONDITIONS AFFECTING THE UPPER AIR PASSAGES.

BY E. C. ROEMELE, M. D., FRANKFORT, KY.

Case I.—E. J., aet 24. Diagnosis: Chronic Nasal Catarrh. Duration, three years. Patient complained of a feeling of fullness in the nares and increase of the secretions, the character being thick and greenish, which dropped posteriorly into the pharynx, causing paroxysms of "hawking" which were more marked in the morning just after arising. The voice had a peculiar nasal intonation, the sense of smell was abolished almost entirely and hearing was impaired, due to the extension of the inflammation into the eustachian tubes. The patient also complained of a constant dull headache. I at once prescribed Glyco-Thymoline and had him use the K. & O. Nasal Douche every four hours, using the Glyco-Thymoline in 25 per cent solution. I directed him to spray his throat with an atomizer, using undiluted Glyco-Thymoline every four hours and also gave him one teaspoonful of Glyco-Thymoline four times a day internally. This was done on account of the catarrhal condition of his stomach. After two weeks the hawking had ceased, his voice took on a more natural tone and hearing and smelling senses were improved. He continued to improve when after fifteen weeks he was entirely cured. There has been no return during the past ten months.

Case II.—Willie Green, aet 7. Diagnosis: Hypertrophy of Tonsils. This case was referred to me by Dr. D., to have his tonsils removed. The doctor stated that he had used every known remedy to reduce them, his last resort being Iodine which he applied in undiluted form, also giving him internal treatment. When I examined his throat I found the tonsils extremely large, so large in fact that the opening was not as large as a slate pencil.

He was a terrible mouth breather and could easily be heard from one room to another. He would not consent to the operation and his mother would not permit us to administer chloroform. I then decided to attempt to cure them without the operation. I prescribed a pound bottle of Glyco-Thymoline and directed the mother to spray his throat thoroughly every three hours with a atomizer. She called in again in one week and the swelling had subsided and the child ceased to breathe as hard as he had breathed. This same treatment was continued. He was returned to my office in three weeks when the tonsils were normal in size; he kept his mouth closed when he slept. The treatment was continued several weeks longer when he was discharged as cured. It has now been eight months and no return whatever of any symptom of the disease.

Case III.—Ella N., aet 27. Diagnosis: Rhino-Pharyngitis. Duration, six years, presenting characteristic symptoms of severe type. Patient had to vomit after each meal on account of hawking the mucus out, which she said would drop into her throat. When she would arise in the morning she would have to hawk and cough half an hour before she would be relieved of the mucus which she said came out of her throat in the shape of round balls. I directed her to use the K. & O. Douche, filling it with Glyco-Thymoline pure, flushing out the nasal cavaties three times a day and directed her to spray her throat with Glyco-Thymoline, one part to one of water, three times a day. Improvement was immediate. After five weeks instead of using the Glyco-Thymoline in the douche in undiluted form she was directed to dilute it with one part of water. After the fourth day she ceased vomiting and hawking. This treatment was continued, however, for four months when she was discharged cured.

---

### CHRONIC CONSTIPATION.

Every physician realizes that there are various and diveres causes for chronic constipation, and that no single remedy or combination of remedies will relieve every patient. A large proportion of cases, however, appear to be due to Hepatic Insufficiency and Biliary Stasis. The natural biliary fluid, when sufficient in quality and thin enough to flow freely into the intestine, acts as a physiologic laxative or natural stimulant to peristalis. *Per contra*, when the bile is not formed and secreted in sufficient quantity, or in its normal condition of fluidity, or *when it is dificient in the natural bile acids*, a more or less obstinate constipation is likely to result. In such cases, the rational remedy is one which encourages the liver to renewed activity, increases and liquefies the biliary flow, augments the proportion of bile acids in the bils, and thus normally regulates bowel movement.

CHOLOGESTIN efficiently exercises this desirable cholagogue action, as it supplies, in palatable and tolerable form, a sufficient dosage of the amorphous salt of the natural bile acid (glycocholic acid) to stimulate hepatic activity, and thus promote *both bile and bowel drainage*. The contained salicylate of sodium (from natural oil of wintergreen) reenforces the cholagogue action of the bile acid salt, and alsco exercises its well-known liquefying effects upon the biliary fluid. To produce appreciable results, Chologestin must be given regularly for some little time, as it is *not an immediate laxative or cathartic*.

Samples, formula and complete literature sent upon request to

F. H. STRONG COMPANY,

58 Warren street, New York.

Page(s) missing

